# Trait anxiety and unplanned delivery mode enhance the risk for childbirth-related post-traumatic stress disorder symptoms in women with and without risk of preterm birth: A multi sample path analysis

**DOI:** 10.1371/journal.pone.0256681

**Published:** 2021-08-31

**Authors:** Sarah Sommerlad, Karin Schermelleh-Engel, Valentina Lucia La Rosa, Frank Louwen, Silvia Oddo-Sommerfeld

**Affiliations:** 1 Department of Gynecology and Obstetrics, University Hospital of Frankfurt, Frankfurt, Germany; 2 Institute of Psychology, University of Frankfurt, Frankfurt, Germany; 3 Department of Educational Sciences, University of Catania, Catania, Italy; Academic College of Tel Aviv-Jaffa, ISRAEL

## Abstract

Childbirth-related post-traumatic stress disorder (CB-PTSD) occurs in 3–7% of all pregnancies and about 35% of women after preterm birth (PTB) meet the criteria for acute stress reaction. Known risk factors are trait anxiety and pain intensity, whereas planned delivery mode, medical support, and positive childbirth experience are protective factors. It has not yet been investigated whether the effects of anxiety and delivery mode are mediated by other factors, and whether a PTB-risk alters these relationships. 284 women were investigated antepartum and six weeks postpartum (risk-group with preterm birth (RG-PB) *N* = 95, risk-group with term birth (RG-TB) *N* = 99, and control group (CG) *N* = 90). CB-PTSD symptoms and anxiety were measured using standardized psychological questionnaires. Pain intensity, medical support, and childbirth experience were assessed by single items. Delivery modes were subdivided into planned vs. unplanned delivery modes. Group differences were examined using MANOVA. To examine direct and indirect effects on CB-PTSD symptoms, a multi-sample path analysis was performed. Rates of PTS were highest in the RG-PB = 11.58% (RG-TB = 7.01%, CG = 1.1%). MANOVA revealed higher values of CB-PTSD symptoms and pain intensity in RG-PB compared to RG-TB and CG. Women with planned delivery mode reported a more positive birth experience. Path modeling revealed a good model fit. Explained variance was highest in RG-PB (*R*^2^ = 44.7%). Direct enhancing effects of trait anxiety and indirect reducing effects of planned delivery mode on CB-PTSD symptoms were observed in all groups. In both risk groups, CB-PTSD symptoms were indirectly reduced via support by medical staff and positive childbirth experience, while trait anxiety indirectly enhanced CB-PTSD symptoms via pain intensity in the CG. Especially in the RG-PB, a positive birth experience serves as protective factor against CB-PTSD symptoms. Therefore, our data highlights the importance of involving patients in the decision process even under stressful birth conditions and the need for psychological support antepartum, mainly in patients with PTB-risk and anxious traits.

**Trial registration number:**NCT01974531 (ClinicalTrials.gov identifier).

## Introduction

For expectant parents, childbirth is a joyous but also challenging event. Previous research has already provided evidence that women are more vulnerable to develop mental health problems during the peripartum [1].

In this regard, considerable research attention has been paid to the trauma of birth. Indeed, since 1994, the Diagnostic Statistical Manual (DSM) included childbirth as a potentially traumatic event [2]. “Birth trauma” has been defined as an event occurring during labour and birth that may be a serious threat to the life and safety of the mother and/or child [2]. De Graaff et al. [3] showed that 9 to 44% of all mothers report at least traumatic aspects of the delivery situation, even if the symptoms significantly decrease within the first weeks after birth in the majority of women [4].

The experience of a real or perceived trauma during labor and birth can be associated with different and more or less long-lasting consequences. The most serious consequence is surely the development of a childbirth-related post-traumatic stress disorder (CB-PTSD) with symptoms of reexperiencing, avoidance, negative cognitions and mood, hyperarousal [5]. The prevalence of CB- PTSD varies significantly, according to recent studies. For example, a review article by Grekin and O’Hara [6] reported a prevalence rate of 3.17% while, according to Yildiz et al. [7], 4% of women develop CB-PTSD symptoms, which may or may not evolve into CB-PTSD diagnosis.

CB-PTSD symptoms are associated with potentially lasting and negative consequences for the well-being of women, infants and families. Indeed, several studies underlined that CB-PTSD symptoms may affect the parent–infant bond, child’s attachment, self-image and emotions, and social relationships [8–10]. Furthermore, Parfitt & Ayers [11] investigated the impact of CB-PTSD symptoms on the quality of couple relationship, showing that it was associated with a worse relationship. However, this association was fully mediated by the presence of depressive symptoms.

It is important to underline that postpartum PTSD may be a direct consequence of a birth experienced as traumatic but may also be a continuation of pre-existing PTSD, a reactivation of a PTSD that had previously remitted, or a consequence of an event unrelated to childbirth [12].

Given the confusion in the use of terminology in the literature, this study refers to postpartum PTSD symptoms related to the traumatic experience of childbirth, excluding postpartum PTSD.

CB-PTSD is associated with obstetric factors as well as with psychological risk factors. Recent research suggests that risk factors for traumatic birth experience include previous psychopathology, pregnancy-related pathology, perceived lack of support by obstetric caregivers, and several types of negative feelings concerning birth [1, 3]. Previous findings also indicate a strong relationship between trait anxiety and the genesis of CB-PTSD symptoms [6, 13–17]. Thus, trait anxiety is regarded as a risk factor for the development of PTSD symptoms in the postpartum period.

Research on protective factors focuses on the quality of support provided by the medical staff, whereby medical support was defined as caregiver support [18–20]. Some studies found that positive support by obstetric staff is a protective factor associated with lower CB-PTSD symptoms [6, 18, 21, 22]. It was shown that a positive perception of obstetric support during labour, regardless of the mode of delivery, can have a protective effect on postpartum mental health [6, 23]. Using a path model with repeated measures over two time points, Gürber et al. [19] analyzed the impact of perceived support by obstetric caregivers on acute stress reactions and depressive symptoms one week and three weeks after birth. They were able to show that a positive perception has an advantageous effect on birth experience and a protective effect against the development of acute stress reactions after birth. However, no further psychological factors were investigated. Additionally, subjective pain intensity was also identified as a significant influencing factor. A low level of subjective pain intensity was found to be a protective factor for CB-PTSD symptoms [6, 14, 16, 24].

Moreover, few studies have shown that CB-PTSD symptoms are associated with the delivery mode. Unplanned birth procedures seem to enhance the risk of CB-PTSD. Previous studies provided evidence that PTSD-risk was highest in women who had unplanned caesarean section or vaginal instrumental delivery [18, 25, 26]. More specifically, these women more frequently report somatization, obsessive compulsive, depression, and anxiety symptoms [25]. Furthermore, it was shown that women experienced stronger feelings of loss of control and helplessness if delivery mode was not the planned one, e.g., unplanned caesarean section or instrumental vaginal delivery. In this regard, the literature on the topic classifies the mode of delivery as planned (elective caesarean section or vaginal delivery) or unplanned (vacuum extraction, emergency cesarean section, or non-emergency caesarean section) [27]. More specifically, it has been shown that women who had a planned delivery perceived their childbirth experience more positively than women who delivered by unplanned interventions [27]. Therefore, the planned delivery mode can be considered a further protective factor against developing CB-PTSD symptoms [18, 28, 29]. Therefore, a high level of perceived control during birth procedure seems to be an important protective factor [14, 28, 30, 31]. Furthermore, preterm birth or at least an initial prematurity risk during pregnancy is a major risk factor for postpartum PTSD [6, 7, 32, 33]. In this regard, at least 35% of mothers after preterm birth (PTB) meet the diagnostic criteria of an acute stress reaction in the first 3–5 days after delivery [34].

Previous studies on CB-PTSD in the context of preterm birth did not differentiate between women with imminent PTB who actually had either a premature birth or who actually had a term birth as the final outcome. Our study aims to investigate a multifactorial explanatory model for CB-PTSD symptoms differentiating between these groups.

Thus, the main goal of this study was to determine the influencing factors for CB-PTSD symptoms in women with prior risk for PTB and to further differentiate between women at risk (risk group, RG) with actual PTB (RG-PB) or actual term birth (RG-TB), and to compare these groups with women of a control group who had a term birth without any prior risk of PTB (CG).

Additionally, we investigated direct and indirect effects of the risk factor trait anxiety and the protective factor planned delivery mode on CB-PTSD symptoms via intervening variables (support by medical staff, pain intensity, positive subjective birth-experience) in the three groups.

We hypothesized that women with imminent preterm birth are at higher risk for CB-PTSD symptoms than healthy controls and that especially women with actual preterm birth (RG-PB) would be emotionally strained, followed by women of the RG-TB. Additionally, we assumed that high levels of the risk factor trait anxiety may enhance CB-PTSD symptoms directly or indirectly by increasing pain intensity as well as reducing positive birth experience and support by medical staff. On the other hand, planned delivery mode as a protective factor may reduce CB-PTSD symptoms directly or indirectly by increasing positive birth experience and the feeling of being supported by medical staff as well as by reducing pain intensity.

## Materials and methods

### Study sample

As part of a longitudinal research project on development of peripartum mental illness in association with the risk of preterm birth, pregnant women were recruited at a German University Hospital. Women with a prior risk of preterm birth were recruited into the risk group (RG) and those without imminent preterm birth into the control group (CG). Recruitment took place between 2013 and 2018. The RG consisted of women who had to be hospitalized between the 24+0 and 36+6 week of pregnancy with a risk of premature birth. Relevant symptoms were shortening of the cervix, premature labour, premature rupture of the membrane. Women of the RG were contacted by staff of the psychology department after being hospitalized with risk of preterm birth. Risk of preterm birth was assessed by the ward physicians. The CG consisted of women with regular pregnancy in outpatient care. They were approached at registration for birth at our hospital around 36 gestational weeks. The RG was retrospectively subdivided into women with an actual preterm birth (RG-PB) and women with an initial prematurity risk, but term birth (RG-TB). We defined these risk groups because we wanted to investigate whether actual preterm birth or the risk of imminent preterm birth leads to higher psychological distress and lower mental well-being. The effects of imminent preterm birth on the expecting mother’s mental health are only seldom studied so far [35].

**Fig 1:**
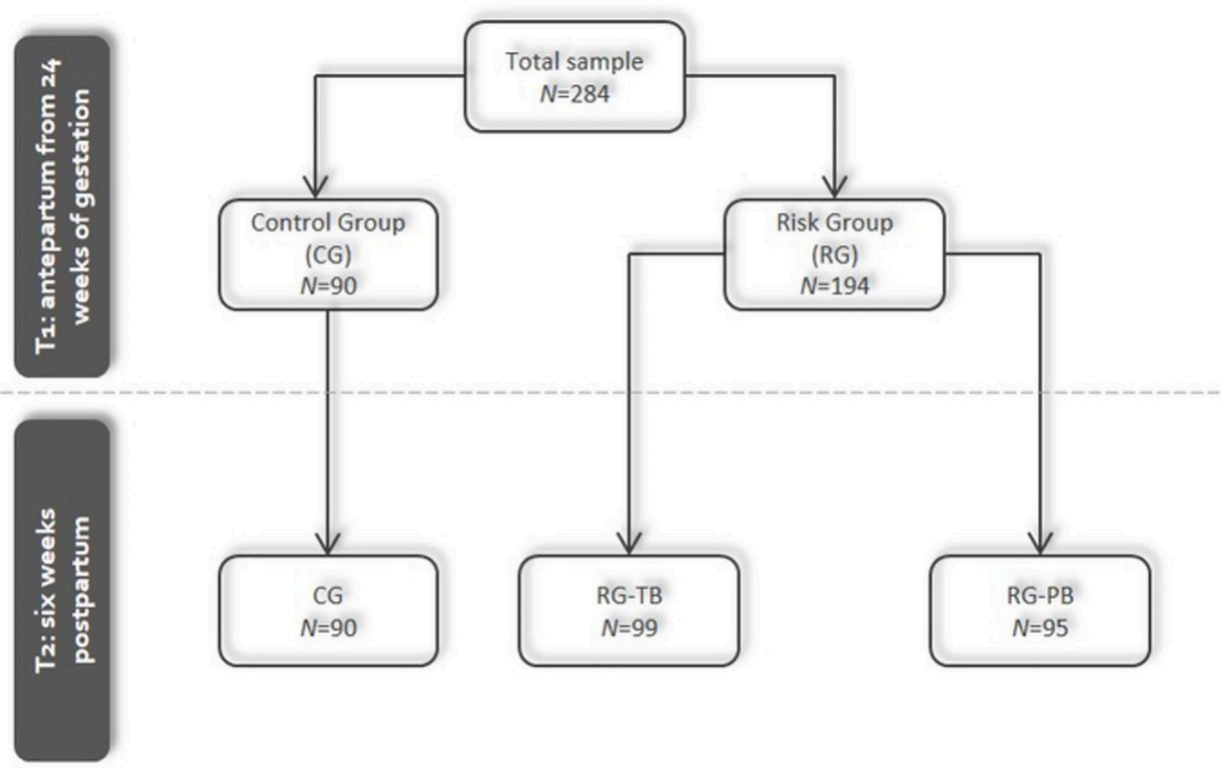
Flowchart of measurement time points and sample sizes of all subgroups.

Women were measured at four-time points. For this publication, only the first two measurement points (late second or third trimester of pregnancy and six weeks postpartum, see Fig 1) were considered, as the direct impact of antepartum and birth-related factors on postpartum CB-PTSD symptoms was investigated. The later measurement timepoints T3 and T4 (6 months and 2 years postpartum) will therefore be considered in a separate article. Inclusion criteria were good knowledge of the German language, a minimum age of 18 years, and a gestational week of at least 24. Furthermore, women with twin pregnancies were excluded from this analysis. At T1, a total of *N* = 446 women participated. However, since only women with complete questionnaires at T1 and T2 were considered for this analysis, sample size was reduced to *N* = 284 women after taking into account all inclusion and exclusion criteria. A dropout analysis was subsequently calculated. For this purpose, correlations were calculated using Kendal’s tau. Due to the unequal distribution of individuals across categories, the bootstrap approach was used instead of the normal standard error. No significant correlations with the group variable (dropout/non-dropout) were observed for the following variables: previous preterm birth or miscarriage, partnership status and duration, multipara, and in vitro fertilization. Dropouts were slightly younger than the sample (r = -.11, p = .01), had a slightly lower level of education (r = -.12, p = .01), and a marginally lower gestational age at recruitment (r = -.14, p< = .01), and at birth (r = -.12, p = .01). However, it should be emphasized that the significant correlations were consistently very low, with an r < .15, so it can be assumed that the groups are comparable.

Participation was voluntary, and all participants gave informed written consent prior to their inclusion in the study. Ethical approval was obtained from the university’s Institutional Review Board (#391–12).

### Instruments

The *State-Trait Anxiety-Depression Inventory* (*STADI* [36]) is a 40-item self-report instrument which aims to distinguish between anxiety and depression while differentiating between temporary (state) and chronic (trait) conditions. The rating scale consists of a 4-point Likert-type scale ranging from 1 (*not at all*) to 4 (*very much*). The STADI has a 4-factor structure of anxiety and depression including emotionality, worry, dysthymia, and anhedonia.

In the present study we used the trait anxiety scale (STADI-T) consisting of two subscales Emotionality (5 items) and Worry (5 items). Reliability of the scale was sufficiently high in our sample with ω = .86 for Emotionality, ω = .87 for Worry, and ω_H_ = .75 for the total scores based on a bifactor model [36]. As the STADI does not yet comprise any cut-off scores, we used the classification in terms of elevated T-values as suggested by the authors based on a representative age and gender-matched sample: scores ≥ 22 correspond to a T*-*value of ≥ 61 (elevated scores) and scores ≥ 32 are equal to T-values of ≥ 70 (extremely high anxiety scores).

To measure CB-PTSD symptoms six weeks postpartum, a birth-related, adapted version (*IES-G* [37]) of the *Impact of Event Scale* (*IES-R* [38]) was used. Sample items are “I think about the birth, even if I don’t want to think about it” and “I feel like it was not true or that it did not happen at all”. The *IES-G* captures the two facets *Intrusion* (7 items) and *Avoidance* (8 items) that may also be used as a single sum score for post-traumatic stress symptoms. All 15 items are rated on a 5-point Likert-type scale ranging from 0 (*never*) to 4 (*often*). Reliability of the scale was sufficiently high in our sample with ω = .75 for Intrusion, ω = .79 for Avoidance, and ω_H_ = .84 for the total scores based on a bifactor model. High risk for PTS corresponds to *IES-G* sum scores ≥ 20. It should be noted that the *IES-G* does not qualify for a diagnosis of PTSD, as it only captures postpartum CB-PTSD symptoms. High risk for PTSD corresponds to *IES-G* sum scores ≥ 20 [37].

In addition to these questionnaires, anamnesis was collected at each time point, including sociodemographic data, pregnancy-related aspects, and birth-related characteristics. We used an 11-point single item rating scale to capture *subjective pain intensity* (“How much pain did you have?”, ranging from 0 (no pain) to 10 (unbearable)) and *birth experience* (“How positive did you experience your birth?”, ranging from 0 (absolutely not positive) to 10 (absolutely positive)), and *support by medical staff* was assessed using a seven-point rating scale (“My overall experience of the support provided by the obstetric team was positive”, ranging from 0 (not at all) to 6 (absolutely)).

**Fig 2:**
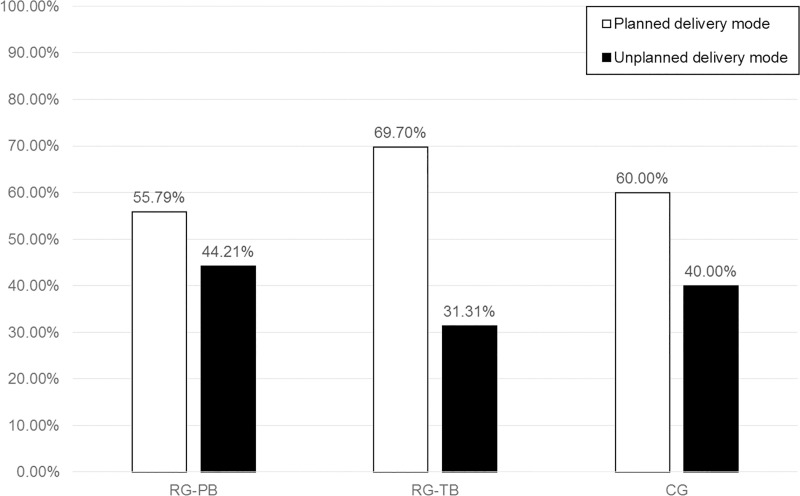
Percentage of women with planned vs. unplanned delivery mode per subgroup.

As mentioned above, delivery modes were subdivided depending on whether they were planned or not (planned = 1, unplanned = 0). Spontaneous vaginal delivery as well as elective caesarean sections constituted the planned delivery mode (1), whereas unplanned caesarean sections, emergency caesarean sections, as well as vaginal operative delivery were assigned to the unplanned delivery mode (0). Fig 2 shows the percentage of women with planned vs. unplanned delivery mode per subgroup.

At T1, *STADI-T* was administered, and at T2, *IES-G* was used. In addition, an anamnesis was obtained at both measurement time points, whereby the variables "*delivery mode*", "*subjective pain intensity*", "*support by medical staff*," and "*birth experience*" were recorded postpartum at T2.

### Statistical analysis

A two-factorial MANOVA using the statistic program *SPSS* version 20 was used to investigate whether the means of the observed variables trauma, pain intensity, birth experience, and support by medical staff differ significantly between the groups RG-PB, RG-TB, and CG, and the type of delivery mode (planned vs. unplanned). Although the assumptions of variance homogeneity and normally distributed variables were violated, MANOVA was nevertheless carried out because Wilks’s lambda is known to be robust against the violation of the normality assumption and equality of covariance matrices if sample sizes are about equal [39, 40].

In order to check whether power had been sufficiently high for detecting group differences, post-hoc power analyses were performed using the G*power program, version 3.1.9.2 [41]. The results showed that for a medium effect size of f^2^(V) = 0.0625, *N* = 284, two factors with 3x2 factor levels, and alpha set at .05, power was 99.6% indicating that a small to medium effect size could have been detected with our sample size.

We conducted a multi-sample path analysis in order to examine direct and indirect effects in all groups for the prediction of CB-PTSD symptoms. For parameter estimation, the robust maximum likelihood estimator of the M*plus* program [42] was used. This estimator uses full information maximum likelihood for handling missing data and takes non-normality of the data into account. Model fit was evaluated using the *χ*^*2*^ test and its associated *p*-value, the root mean square error of approximation (RMSEA), the comparative fit index (CFI), and the standardized root mean square residual (SRMR). Good model fit was indicated by a nonsignificant *χ*^*2*^-value, RMSEA ≤.06, CFI ≥.95, and SRMR ≤.08 [43, 44].

The amount of missing data was very small (0.26% at T1 and 0.78% at T2), and simultaneously replaced when estimating model parameters, using the MLR estimator of the *Mplus* program. For analyses in SPSS, missing values were replaced using multiple imputation.

## Results

### Participant characteristics

Table 1 reports sociodemographic and obstetric characteristics of study participants in the three subgroups. The sociodemographic characteristics were similar between groups. We found a higher proportion of spontaneous vaginal delivery in the RG-TB and in the CG (53.54% and 51.11%, respectively) compared to the RG-PB (31.58%). The percentage of caesarean sections was higher in the risk-group with preterm birth (52.63%) compared to the other two samples (RG-TB: 15.15%; CG: 24.44%). Finally, instrumental vaginal delivery was more prevalent in the risk-group with term-birth and in the control group (31.31% and 24.44%, respectively) than in the risk-group with preterm birth (10.52%).

**Table 1:** Sociodemographic and obstetric characteristics of study participants by subgroups.

	RG-PB (*N* = 95)	RG-TB (*N =* 99)	CG (*N* = 90)
	*N*(%) or *M*±*SD*	*N*(%) or *M*±*SD*	*N*(%) or *M*±*SD*
**Sociodemographic characteristics**			
**Age**	33.51±4.35	33.50±4.40	32.58±3.71
**Level of education^a^**:			
**Secondary school or less**	29 (30.53%)	34 (34.34%)	23 (25.56%)
**High school**	45 (47.37%)	43 (43.43%)	47 (52.22%)
**College**	20 (20.05%)	19 (19.19%)	20 (21.88%)
**Relationship status**			
**Single**	2 (2.11%)	2 (2.08%)	0 (0%)
**In a relationship**	93 (97.89%)	96 (97.96%)	90 (100%)
**Married**	68 (71.58%)	73 (74.49%)	68 (75.56%)
**Obstetric characteristics**			
**Gestation week**			
**when recruited**	28.84 ±3.29	29.95±3.55	35.00±1.57
**at delivery**	32.92±3.08	38.62±1.31	39.71±1.14
**Duration of in-patient stay (before birth)**	27.14±25.71	19.80±21.11	4.29±5.24
**Parity**			
**Primipara**	66 (69.47%)	68 (68.69%)	57 (63.33%)
**Multipara**	29 (30.53%)	31 (31.31%)	33 (36.67%)
**Prior pregnancies**			
**Preterm birth**	11 (11.58%)	9 (9.09%)	3 (3.33%)
**Miscarriage**	22 (23.15%)	20 (20.20%)	8 (8.89%)
**In-vitro-fertilization**	10 (10.53%)	14 (14.14%)	3 (3.30%)
**Mode of delivery:**			
**Spontaneous vaginal delivery**	30 (31.58%)	53 (53.54%)	46 (51.11%)
**Caesarean section**	55 (57.89%)	31 (31.31%)	22 (24.44%)
**elective**	23 (24.21%)	16 (16.16%)	8 (8.88%)
**secondary**	32 (33.68%)	15 (15.15%)	14 (15.56%)
**Instrumental vaginal delivery**	10 (10.52%)	31 (31.31%)	22 (24.44%)
**BMI**	24.90±6.11	24.03±4.75	23.57±4.46

RG-PB = risk-group with preterm birth; RG-TB = risk-group with term-birth; CG = control-group; N = Study sample; M = Median; SD = standard deviation; BMI = Body Mass Index;

^a^ four participants did not answer the item.

### Percentage of women with high risk of CB-PTSD symptoms

The percentage of women with high risk for CB-PTSD symptoms (*IES-G* ≥ 20) was 6.7% in the total sample. As expected, the prevalence was highest in the RG with preterm birth (11.58%) compared to RG-TB (7.01%) and CG (1.1%).

### Group differences between women with and without risk of PTB

To investigate whether the means of CB-PTSD symptoms, subjective pain intensity and subjective birth experience differ significantly between the groups (RG-PB, RG-TB, and CG) as well as between modes of delivery (planned and unplanned delivery), a two-factorial MANOVA was performed.

Using Wilks’ lambda, there was a significant overall effect of group on birth experience, pain intensity, and CB-PTSD symptoms six weeks postpartum, Λ = .84, *F*(6, 526) = 7.79, *p*≤.01, and a significant effect of delivery mode on the observed dependent variables, Λ = .82, *F*(3, 263) = 19.67, *p*≤.01. The follow-up ANOVAs revealed significant group differences between the groups RG-PB, RG-TB, and CG regarding CB-PTSD symptoms six weeks postpartum (*F*(2, 265) = 3.34, *p* = .04, *η*_p_^2^ = .03) and pain intensity (*F*(2, 265) = 15.85, *p*≤.01, *η*_p_^2^ = .11).

**Fig 3:**
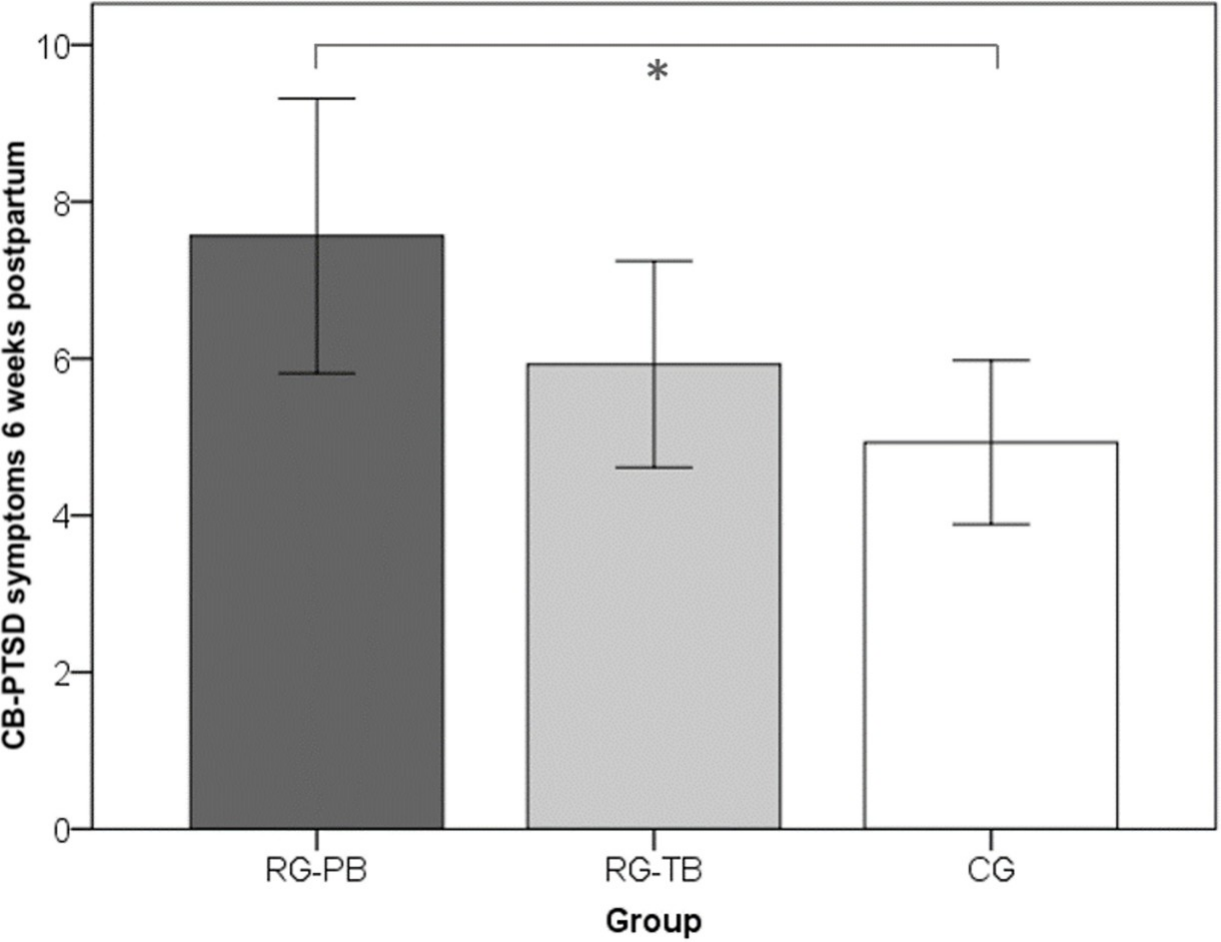
Bar graphs for each of the observed significant mean differences (CB-PTSD symptoms). (A) CB-PTSD symptoms six weeks postpartum. RG-PB = risk-group with preterm birth; RG-TB = risk-group with term-birth; CG = control-group. Error bars represent 95% confidence intervals.

**Fig 4:**
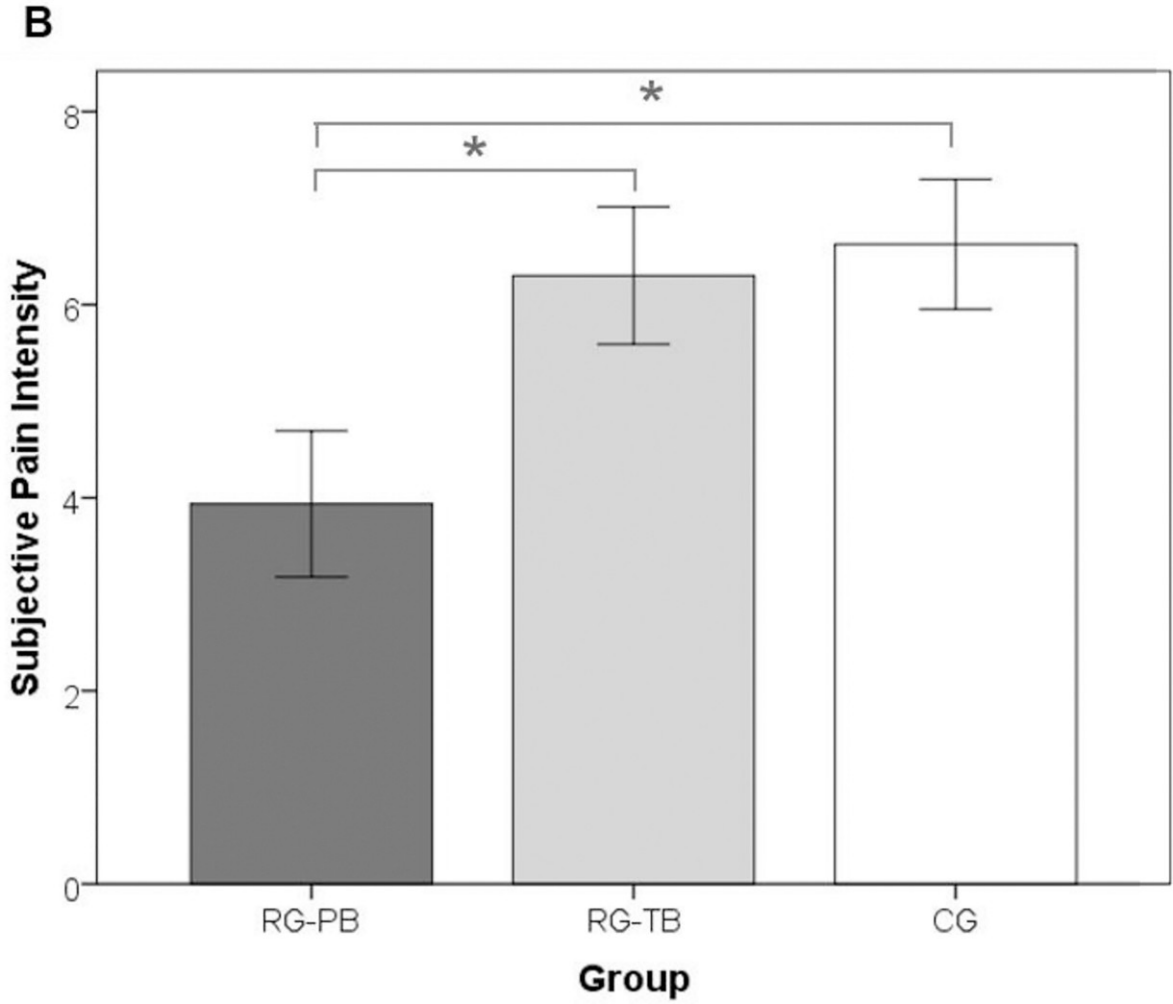
Bar graphs for each of the observed significant mean differences (pain intensity). (B) Subjective pain intensity. RG-PB = risk-group with preterm birth; RG-TB = risk-group with term-birth; CG = control-group. Error bars represent 95% confidence intervals.

**Fig 5:**
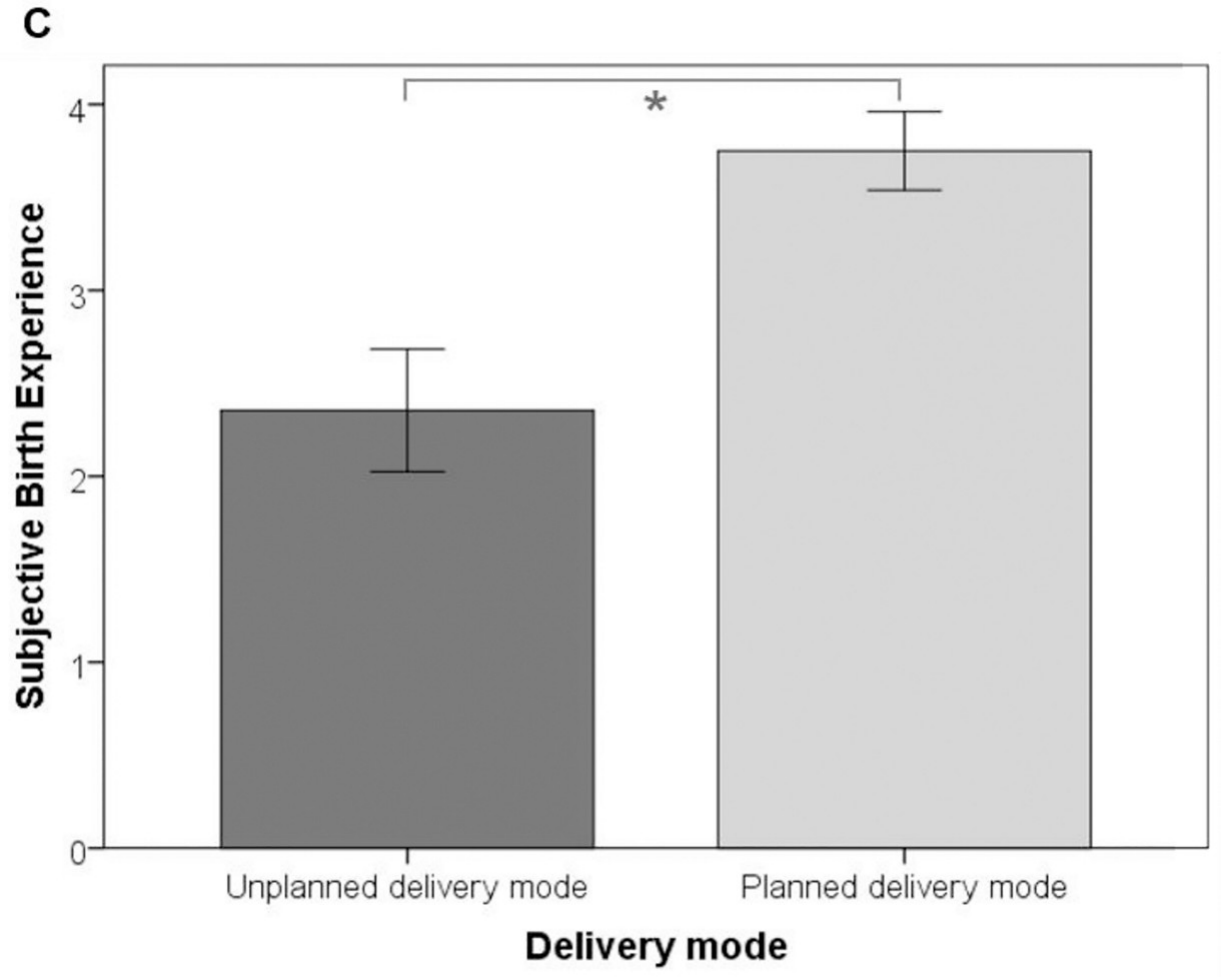
Bar graphs for each of the observed significant mean differences (subjective birth experience). (C) Subjective birth experience. RG-PB = risk-group with preterm birth; RG-TB = risk-group with term-birth; CG = control-group. Error bars represent 95% confidence intervals.

As expected, CB-PTSD symptoms were highest in RG-PB and lowest in CG (Fig 3). Pain intensity was lowest in women with preterm birth, while RG-TB and CG did not differ significantly (Fig 4). Additionally, significant mean differences were observed between women with planned and unplanned delivery mode regarding subjective birth experience (*F*(1, 265) = 49.27, *p*≤.01, *η*_p_^2^ = .18): Women with planned delivery mode reported a more positive birth experience than women with unplanned delivery mode (Fig 5). There was no significant interaction effect between the factors group and mode of delivery for any of the considered dependent variables, Λ = .98, *F* (6, 526) = .87, *p =* .52.

### Multi-group path analysis for prediction of CB-PTSD symptoms

**Fig 6:**
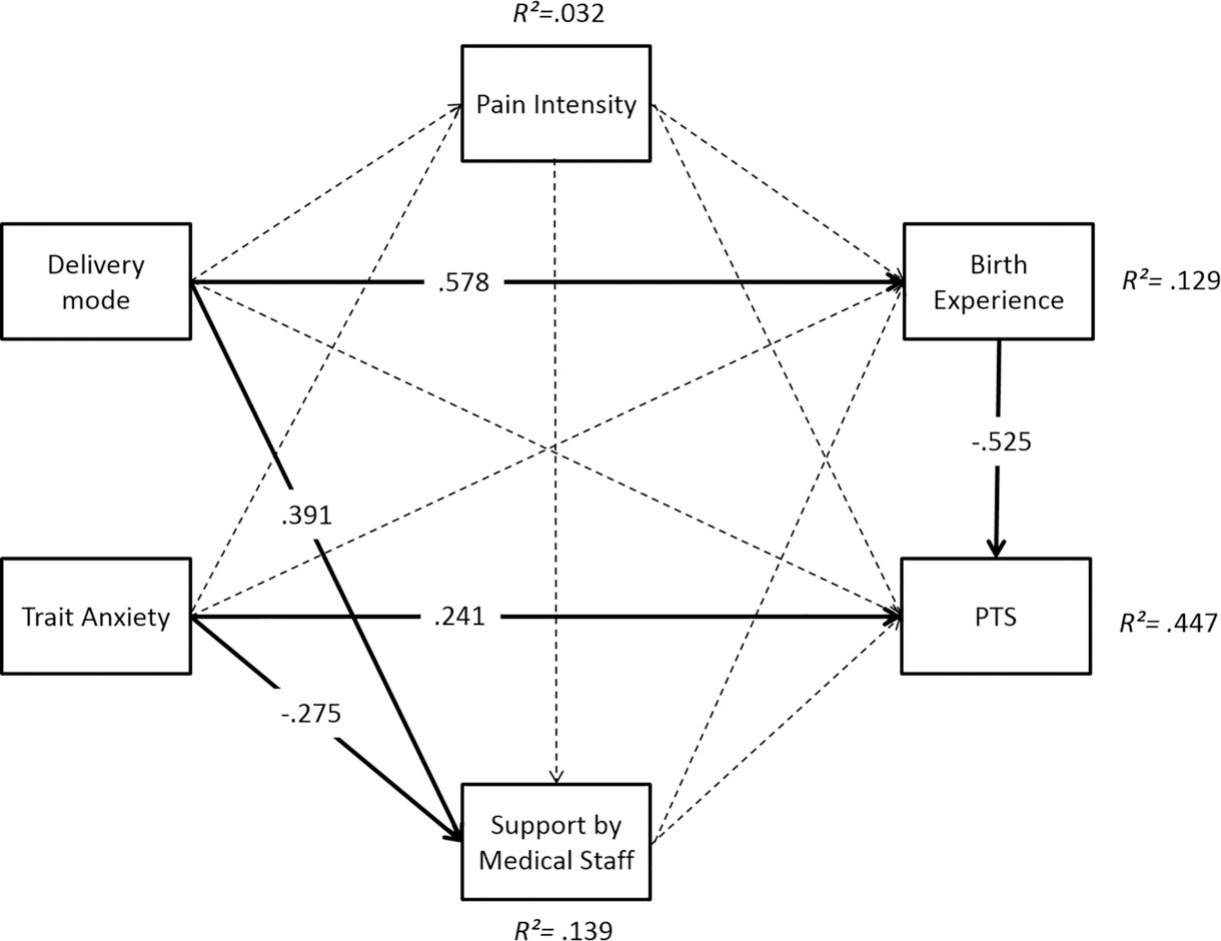
Path model for prediction of CB-PTSD symptoms 6 weeks postpartum in the risk group with preterm birth (RG-PB). Significant paths (*p* < .01) are represented by solid lines, non-significant paths by dashed lines.

**Fig 7:**
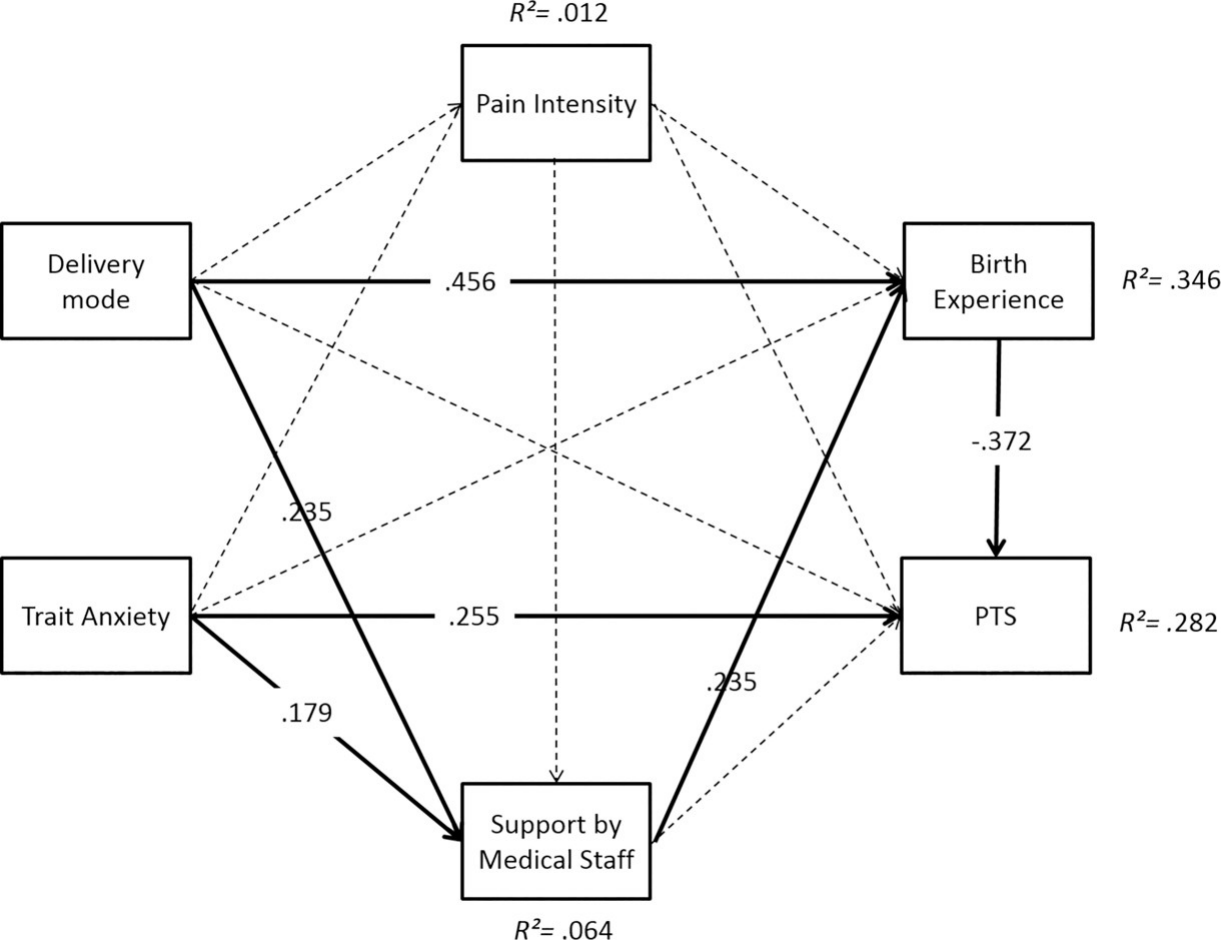
Path model for prediction of CB-PTSD symptoms 6 weeks postpartum in the risk group with term birth (RG-TB). Significant paths (*p* < .01) are represented by solid lines, non-significant paths by dashed lines.

**Fig 8:**
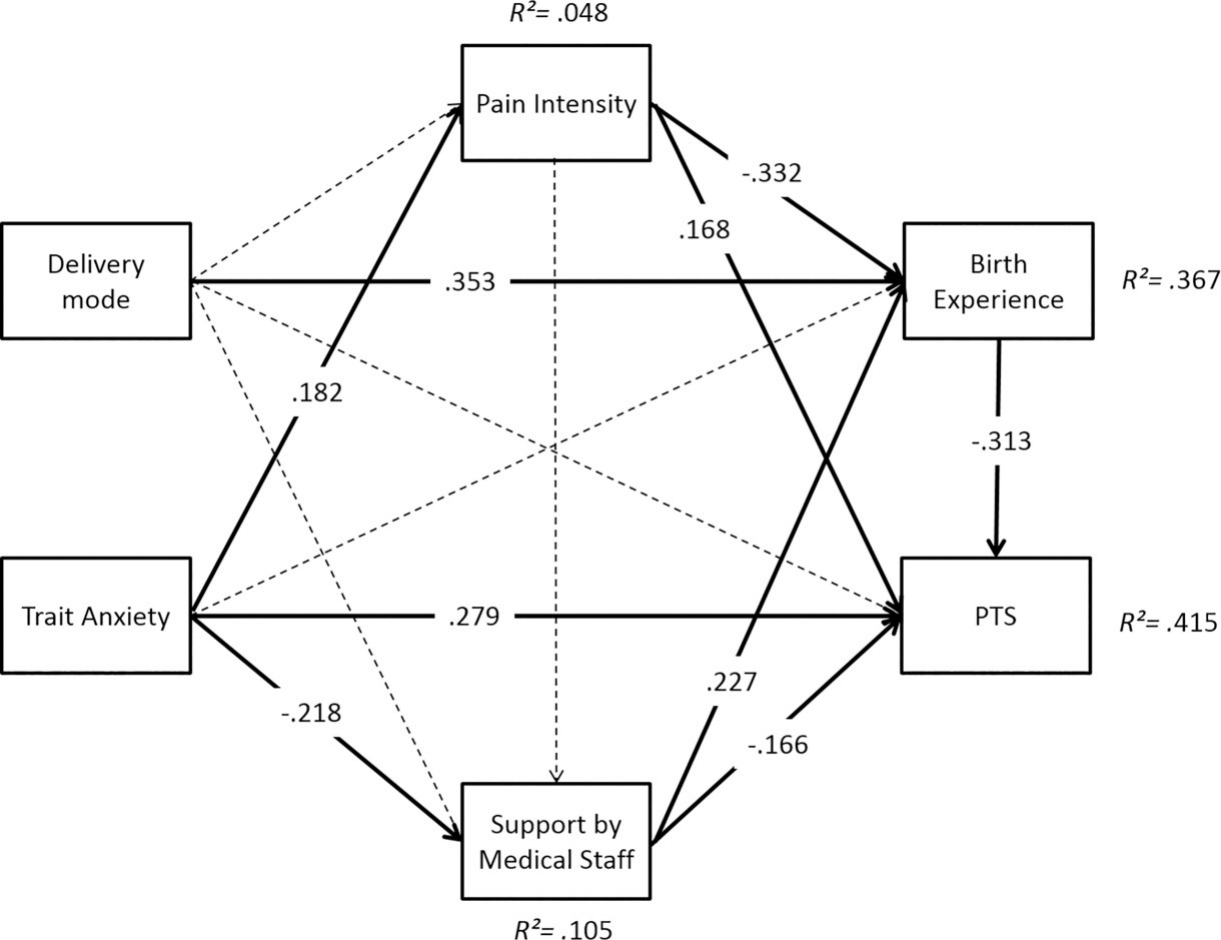
Path model for prediction of CB-PTSD symptoms 6 weeks postpartum in the control group (CG). Significant paths (*p* < .01) are represented by solid lines, non-significant paths by dashed lines.

The multi-group path analysis revealed a good model fit (*χ*^*2*^ = 0.35, *df* = 2, *p* = .84, *RMSEA* = .00, *CFI* = 1.00, *SRMR* = .01). Results partially differed between the groups. Overall, the path model accounted for *R*^*2*^ = 44.7% of variance of CB-PTSD symptoms 6 weeks postpartum in the RG-PB, *R*^2^ = 28.2% in the RG-TB, and *R*^2^ = 41.5% in the CG (Figs 6–8).

In all groups, trait anxiety directly increased CB-PTSD symptoms (RG-PB: *β* = .241; RG-TB: *β* = .255; CG: *β* = .279; all *p*s*≤* .01). An indirect effect of trait anxiety via subjective birth experience was observed in the RG-TB (*β* = -.079, *p =* .033).

Delivery mode did not directly influence CB-PTSD symptoms in any group. However, the planned delivery mode indirectly reduced CB-PTSD symptoms via birth experience in each group (RG-PB: *β* = -.151, *p* = .021; RG-TB: *β = -*.169, *p* = .014; CG: *β =* -.110, *p* = .023).

A positive birth experience directly reduced CB-PTSD symptoms in all groups (RG-PB: *β =* -.525; RG-TB: *β =* -.369; CG: *β =* -.313; all *p*s*≤*.01), while a higher pain intensity directly enhanced CB-PTSD symptoms only in the CG (*β =* .168, *p* = .035). Furthermore, pain intensity indirectly enhanced PTS via birth experience in the CG (*β =* .104, *p =* .035). Medical support directly reduced CB-PTSD symptoms in the CG (*β =* .168, *p≤* .01), but not in the risk groups. CB-PTSD symptoms were indirectly reduced by medical support via birth experience in the CG (*β =* -.079, *p =* .033).

## Discussion

In the present study, we investigated CB-PTSD symptoms in women with imminent preterm birth who either had a preterm birth (RG-PB) or a timely birth (RG-TB) compared to a control group of women without the risk of preterm birth (CG). Group differences revealed that the RG-PB exhibited higher scores in trauma and pain intensity than the other groups. Differentiating between delivery modes (planned vs. unplanned) showed that women with a planned delivery mode reported a more positive birth experience than women with an unplanned delivery mode.

Using multi-sample path modelling, direct and indirect longitudinal effects of delivery mode and trait anxiety on CB-PTSD symptoms six weeks postpartum by taking into account obstetric and psychological factors (subjective birth experience, pain intensity, and support by medical staff) were investigated. Compared to Gürber et al. [19], who also used a path analysis in their study, our analysis was characterized in particular by the differentiation of the risk group into subgroups with and without preterm birth and by differentiating between planned and unplanned delivery modes.

For all three groups we could show that trait anxiety was a direct risk factor and planned delivery mode an indirect protective factor for CB-PTSD symptoms. However, the three groups differed with regard to the indirect effects of these factors on CB-PTSD symptoms transmitted by other protective factors. For example, in the CG, trait anxiety enhanced CB-PTSD symptoms indirectly through reducing support of medical staff, whereas perceived support additionally reduced PTS directly and indirectly via birth experience. These effects were not found in both risk groups.

As our results indicate, higher levels of antepartum trait anxiety are associated with higher CB-PTSD symptoms values six weeks postpartum. These results are in accordance with the previous research [13, 15, 22, 45]. We showed that trait anxiety is a determinant of CB-PTSD symptoms independent of the group of pregnant women. While it has been state-of-the-art in the past that traits are stable over time, recent research indicates that traits can be altered by environmental influences, development within persons, or by clinical interventions [46]. Therefore, in order to identify anxious women at an early stage, antenatal psycho-diagnostic screening in the outpatient obstetrics area should be considered.

In the RG-TB, the planned delivery mode indirectly reduced CB-PTSD symptoms by enhancing support and positive birth experience. Especially in the RG-PB, positive birth experience is an important protective aspect against CB-PTSD symptoms. This allows to conclude that especially women with imminent preterm birth may reduce their risk of CB-PTSD symptoms if anxiety is treated in time and if the delivery mode matches with the planned mode and if mothers feel supported by medical staff. These results furnish evidence in how far a good medical support by the obstetricians can influence the psychological outcome of women after birth. Although reasons for not being able to fulfil the desired delivery mode of mothers are complex and the process of labouring cannot be controlled to a maximum extend by the becoming mother and also by the obstetricians or midwives, the present results implicate that a good communication regarding women’s intentions of delivery mode before and after birth are essential factors for the mental health of mothers.

Another interesting result refers to sufficient pain control during childbirth as being a protective factor only in the CG. These findings are consistent with results from former studies [6, 14, 16, 20]. A somewhat unexpected result in our data was that subjective pain intensity was lowest in both risk groups. An explanation could be that mothers in the RG-PB are so involved in caring for their preterm born infants and are very concerned about the baby’s health at the same time, and therefore do not focus on their own pain. In the RG-TB, on the other hand, mothers may be so relieved by a term birth after the prolonged threat of premature birth, that it may be easier for them to get over the pain. It is also important to mention that women in the RG-PB had the highest rate of caesarean section. Due to peridural anaesthesia during the caesarean section, women of course are not in pain during the main birth procedure. Thus, pain perception does not set in until the anaesthesia wears off. As subjective pain intensity was assessed only six weeks postpartum and not immediately after birth there may be a memory-related bias in our data. Therefore, it should be critically noted that pain intensity should be assessed earlier after birth in future research to avoid such adverse effects.

Some further limitations should also be noted. First, although the use of single-item measures in empirical research should be taken with caution [47], single-item measures are easy to administer and less time-consuming than multi-item scales, an advantage especially for postpartum women who are already experiencing high levels of distress. Rather than omitting potentially relevant constructs and therefore risking misspecification of the model, we included single-item measures of the constructs *subjective pain intensity*, *birth experience*, and *support by medical staff*. While subjective pain intensity is often captured by a single item, the psychometric properties of the other two measures should to be investigated in the future.

Second, particularly in the RG-TB, there seem to be further influencing factors that have not yet been considered in our analyses. For example, antepartum traumatization has been identified as a risk factor for CB-PTSD symptoms in previous studies. Screening for antepartum traumatization and dissociative symptoms should be included in future research. Furthermore, the *IES-G* only allows a risk assessment for CB-PTSD symptoms but not a diagnosis. As an alternative method the Posttraumatic Diagnostic Scale (*PDS*) [48] may be used, which may also be suited for a PTSD diagnosis.

Third, it may be a bias that the first measurement point is heterogeneous in the risk group as we assessed patients 1 to 3 days after arriving at our obstetric department. The “gestational week when recruited” did not differ significantly between RG-PB and RG-TB (see Table 1). We could not use separate groups for extremely preterm and very preterm women because of the small sample size for the extremely preterm group in this sample (extremely preterm <28 weeks: 6 persons; very preterm 28–<32 weeks: 19 persons, moderate or late preterm 32–<37: 70 persons. The merged group RG-very PB (including 6+19 = 25 persons) did not differ from the RG-PB group with regard to three dependent variables we used in our article. Therefore, we chose not to divide the RG-PT group into subgroups in this article. However, in future studies, differences between the three risk groups and their effects of on criterion variables should be investigated in more detail using larger sample sizes.

However, the present research could demonstrate the importance of investigating complex relationships between protective and risk factors in order to understand how CB-PTSD symptoms may be reduced by clinical interventions while taking the risk of preterm birth into account.

Although other protective and risk factors could be investigated in future studies, our results highlight that the presented path model already explains a significant amount of the variability in CB-PTSD symptoms in all groups, with more than 40% explained variance in the RG-PB and in the CG.

## Conclusions

Our study shows that the risk of preterm birth–independent of the outcome preterm or term birth—is associated with higher rates of CB-PTSD symptoms in women six weeks after birth compared to the control group with term birth. This finding is supported by the result that the path model partially differed between the groups.

The observed differences between both risk groups and the control group provide practical implications for medical staff involved in peripartum care, as women of the different groups seem to have partially different needs. The focus of subsequent research should therefore be on the investigation of specific interventions and therapeutic measures for post-traumatic stress symptoms in women with and without preterm birth. Further mediator variables should be analysed, although our path model detected a high amount of variability of CB-PTSD symptoms mainly in the specific risk group with PTB and in the control group. Furthermore, our results indicate that anxious women in particular have a significantly increased risk of developing CB-PTSD symptoms. An early psychological screening appears to be useful in order to make psychological support available to these patients at an early stage. Furthermore, our results indicate that a delivery mode, which is mismatched with the intention, may enhance CB-PTSD symptoms, although only indirectly via support by medical staff. Therefore, our data highlight the importance of stronger communication between staff and patients, aiming to include the patients in the decision process even under unplanned, stressful birth conditions.
